# Unraveling immunosenescence in sepsis: from cellular mechanisms to therapeutics

**DOI:** 10.1038/s41419-025-07714-w

**Published:** 2025-05-16

**Authors:** Yanghanzhao Wang, Hao Zhang, Changhong Miao

**Affiliations:** 1 Department of Anesthesiology, Zhongshan Hospital, Fudan University, Shanghai, China; 2 Shanghai Key laboratory of Perioperative Stress and Protection, Shanghai, China; 3 Department of Anesthesiology, Shanghai Medical College, Fudan University, Shanghai, China

**Keywords:** Sepsis, Cell death and immune response

## Abstract

Sepsis is a life-threatening multiple organ dysfunction resulting from a dysregulated host response to infection, and patients with sepsis always exhibit a state of immune disorder characterized by both overwhelming inflammation and immunosuppression. The aging of immune system, namely “immunosenescence”, has been reported to be correlated with high morbidity and mortality in elderly patients with sepsis. Initially, immunosenescence was considered as a range of age-related alterations in the immune system. However, increasing evidence has proven that persistent inflammation or even a short-term inflammatory challenge during sepsis could trigger accelerated aging of immune cells, which might further exacerbate inflammatory cytokine storm and promote the shift towards immunosuppression. Thus, premature immunosenescence is found in young sepsis individuals, which further aggravates immune disorders and induces the progression of sepsis. Furthermore, in old sepsis patients, the synergistic effects of both sepsis and aging may cause immunosenescence-associated alterations more significantly, resulting in more severe immune dysfunction and a worse prognosis. Therefore, it is necessary to explore the potential therapeutic strategies targeting immunosenescence during sepsis.

## Facts

- Sepsis induces premature immunosenescence in young patients, which further results in immune disorders, including excessive inflammation and persistent immunosuppression.
- In aged patients with sepsis, the synergistic effects of both sepsis and aging may cause immunosenescence-associated alterations more significantly, resulting in more severe immune dysfunction and a worse prognosis.
- Therapeutic strategies targeting immunosenescence might be promising in the treatments of sepsis in the future.

## Open questions

- What are the mechanisms underlying the differences in the phenotype and function of immune cells between young septic patients with premature immunosenescence and elderly patients?
- What molecular factors regulate the chemotaxis of aged neutrophils during sepsis?
- What are the mechanisms underlying sepsis-induced premature aging of DCs, NK cells and B cells?
- Whether the treatments targeting immunosenescence, such as thymus regeneration or IL-7 administration, could be used to improve the prognosis of sepsis patients in the future?

## Introduction

Sepsis is characterized by life-threatening organ dysfunction due to the dysregulation of host immune responses [1], and it remains one of the leading causes of death in intensive care units (ICUs) for decades [2]. Specifically, sepsis initiates complicated interactions between host pro-inflammatory and anti-proinflammatory processes [3]. Acute inflammatory cytokine storm contributes to early death in sepsis, and persistent immunosuppression is considered as the major cause of long-term mortality [4]. Additionally, the hallmark of chronic critical illness (CCI) after sepsis is persistent inflammation, immunosuppression, and catabolism syndrome (PICS), which was first proposed in 2012 [5, 6]. Of note, sepsis disproportionally affects the elderly population, with over 60% of sepsis diagnosis occurring in individuals aged 65 years and older [7]. Furthermore, these elderly septic patients typically exhibit a worse prognosis and are more prone to severe complications [8, 9]. This phenomenon can be partly attributed to the aging of immune system, known as immunosenescence [10].

However, several studies have suggested that in young septic individuals, inflammatory stimulation might directly induce accelerated aging of hematopoietic stem cells (HSCs) in bone marrow [11], which is characterized by impaired self-renewal, enhanced myelopoiesis and reduced lymphopoiesis [12, 13]. Moreover, mature immune cells released from bone marrow are also induced to exhibit the senescence associated secretory phenotype (SASP) [14, 15]. The SASP defines the capacity of senescent cells, including immune cells and non-immune cells, to secret a variety of extracellular modulators, such as inflammatory cytokines, chemokines and growth factors [16]. These prematurely aged immune cells can further cause excessive innate immune responses and defective adaptive immune functions, which ultimately result in overwhelming inflammation and the shift to immunosuppressive state in sepsis. Notably, the terms “premature aging” and “senescence” represent related yet distinct cellular states. “Premature aging” refers to immune cells displaying aging or dysfunction before reaching their physiological lifespan, a state that can be induced by infection or inflammation. Conversely, “senescence” describes an irreversible arrest of cellular proliferation resulting from intrinsic aging processes. In elderly patients with sepsis, the combined effects of both sepsis-induced premature aging and pre-existing cellular senescence may cause more significant immunosenescence-associated alterations, resulting in a poorer prognosis [17].

In this review, we start with the introduction of immunosenescence, followed by a discussion of hematopoietic aging in both young and elderly patients with sepsis. Then, we discuss the changes in innate and adaptive immunity during sepsis. In each immune cell type, we discuss the mechanisms underlying sepsis-induced premature aging of immune cells, and explore the impacts on immune dysregulation including excessive inflammation and immunosuppression. We then focus on the distinct phenotypic and functional changes in immune cells in elderly septic patients compared with those in younger patients. Finally, we summarize several potential therapeutic strategies targeting immunosenescence, and provide some therapeutic concepts that could be tested for the treatments of sepsis in the future.

## Immunosenescence and sepsis

Growing evidence demonstrates that both young and elderly sepsis patients undergo immunosenescence, which is closely associated with poor prognosis. In this section, we will first outline the features of immunosenescence, followed by a discussion of its manifestations in both young and elderly sepsis patients.

### Hallmarks of immunosenescence

Aging induces a variety of immune-related alterations, collectively known as “immunosenescence” [18]. The aging of immune system is reported to originate at the apex of the hematopoietic hierarchy, since the senescence of HSCs directly result in the alterations in immune system, namely immunosenescence [19]. HSCs reside in bone marrow, where they generate all types of blood cells and self-renew to maintain the stem cell pool throughout life [20, 21]. During youth, HSCs with balanced production of lymphoid and myeloid cells (bal-HSCs) exceed HSCs with myeloid-biased output (my-HSCs). Therefore, lymphoid cells are promoted to be released and further trigger adaptive immune response, while the release of pro-inflammatory myeloid cells is limited [22]. Hematopoietic aging is characterized by diminished regenerative capacity and skewed differentiation from HSCs (enhanced myelopoiesis and reduced lymphopoiesis) [23]. The mechanisms underlying HSCs aging can be attributed to two main factors. Most causes contributing to HSCs aging result from cell-intrinsic pathways [12], such as DNA damage [24], epigenetic reprogramming [25] and impaired autophagy and mitochondrial activity [26, 27]. Moreover, the aging of bone marrow microenvironment may also result in the impaired HSC function over time [28]. This shift from bal-HSCs to my-HSCs during aging might be a “double-edged sword”. At young age, individuals require adaptive immunity, which is maintained by repeated various pathogens exposure. Since T and B memory cells are capable to survive throughout life, they are enough to exert adaptive immunity to all local pathogens. Consequently, there is no significant benefit to generate fresh T and B cells in the aged stage, while it is of great significance to produce short-lived myeloid cells to defend against acute infections [29]. However, increased production of pro-inflammatory myeloid cells could result in a low-grade and chronic inflammation in aged people, which is called “inflammaging” [30]. And the decreased lymphopoiesis could cause high mortality in elderly adults during a pandemic, such as COVID-19 pandemic [31].

Accumulation of senescent cells in tissues and organs is one of the hallmarks of aging [32]. Upon being released from bone marrow, mature immune cells interact with these aging cells, which further triggers the process of immune cell senescence. During aging, cellular stressors, such as DNA damage, induce senescent cells to accumulate in adipose tissues and liver. And inflammatory mediators secreted by these aging cells further elevate the expression of the NADase CD38 on macrophages, which exhibit a senescent and pro-inflammatory phenotype [33–35]. Moreover, intestinal-barrier permeability increases with aging, contributing to elevated levels of bacterial products in circulation and tissues. These pathogen-associated molecular patterns (PAMPs) can also promote increased expression of CD38 on macrophages [33, 36, 37]. Qiu et al. have demonstrated that of IL-4 expression decreases in several tissues of aged mice, such as liver, muscle and heart. The inhibition of IL-4-STAT6 axis results in DNA damage through suppressing DNA repair genes. And unrepaired DNA is released into cytoplasm, inducing SASP in macrophages via cGAS-STING signaling pathway, which further causes the increased release of IL-1α, TNF, MMP3 and other inflammatory mediators [32].

Additionally, several studies have demonstrated that the aging of thymus, lymph nodes or spleen induces the senescence of lymphocytes [38]. Thymus serves as a primary lymphoid organ crucial for the development of T cells. Age-related thymus involution is one of the hallmarks of immunosenescence characterized by epithelial structure disruption, adipogenesis and thymocyte development arrest [39]. This involution results in impaired T cell development and reduced emigration of naive T cells to the periphery, increased proportion of memory T cells, and a collapse in peripheral T-cell receptor (TCR) repertoire. Moreover, aged lymph nodes are also unable to maintain the balance of naïve T cells [38]. These alterations in the composition and function of the circulating T cell pool cause impaired adaptive immune responses and increased susceptibility to infectious diseases [40, 41]. Additionally, the spleen, body’s largest secondary lymphoid organ, also plays a vital role in adaptive immune responses [42]. The single-cell sequencing (scRNA-seq) data from the spleen suggest that the proportion of T cells declines with age, while the relative amount of plasma cells increases [43]. Impaired antigen capture by B cells has been observed in aged spleens [44].

Overall, immunosenescence originates at the hematopoietic level, characterized by impaired self-renewal capacity and biased differentiation of HSCs. And this process further results in enhanced myelopoiesis and decreased lymphopoiesis. Subsequently, mature immune cells released from bone marrow are induced to exhibit a senescent phenotype through interactions with aged tissues or organs. Ultimately, these changes in immune system cause persistent low-grade inflammation and diminished adaptive immune function.

### Immunosenescence in young patients with sepsis

During acute infection or inflammation, mature immune cells released from bone marrow constitute the first line of defense against pathogens, and work as the main downstream effectors of excessive inflammatory responses [45]. Currently, it has been increasingly acknowledged that immature hematopoietic stem and progenitor cells (HSPCs) are affected by multiple inflammatory mediators [46, 47]. Several studies have proven that HSCs could transiently be activated by inflammatory stimuli from their long-term quiescent state, and then return to their “dormant” state [48, 49].

Previously, mounting evidence has emphasized that acute exposure of inflammatory stimuli does not affect the self-renewal and multi-lineage differentiation capacities of HSCs, while chronic inflammatory stimulation might induce a senescent phenotype featured by myeloid-biased differentiation and decreased self-renewal capacity [50, 51]. For example, a study demonstrates that acute exposure of IL-1 transiently activates myeloid-based differentiation, and it has no effect on HSC self-renewal activity. Additionally, Passegué et al. have proven that acute inflammation could instantly activate autophagy in HSCs from young mice to avoid adverse impacts of inflammatory stimulation, since autophagy play a protective role in aging or aging-related diseases [26, 52]. Moreover, another study also demonstrates that after lipopolysaccharides (LPS) stimulation, myeloid output in young mice returns to baseline within 3 weeks [53]. In contrast, after 20 consecutive days of IL-1 treatment, HSC function and regeneration are severely impaired [54].

**Fig. 1:**
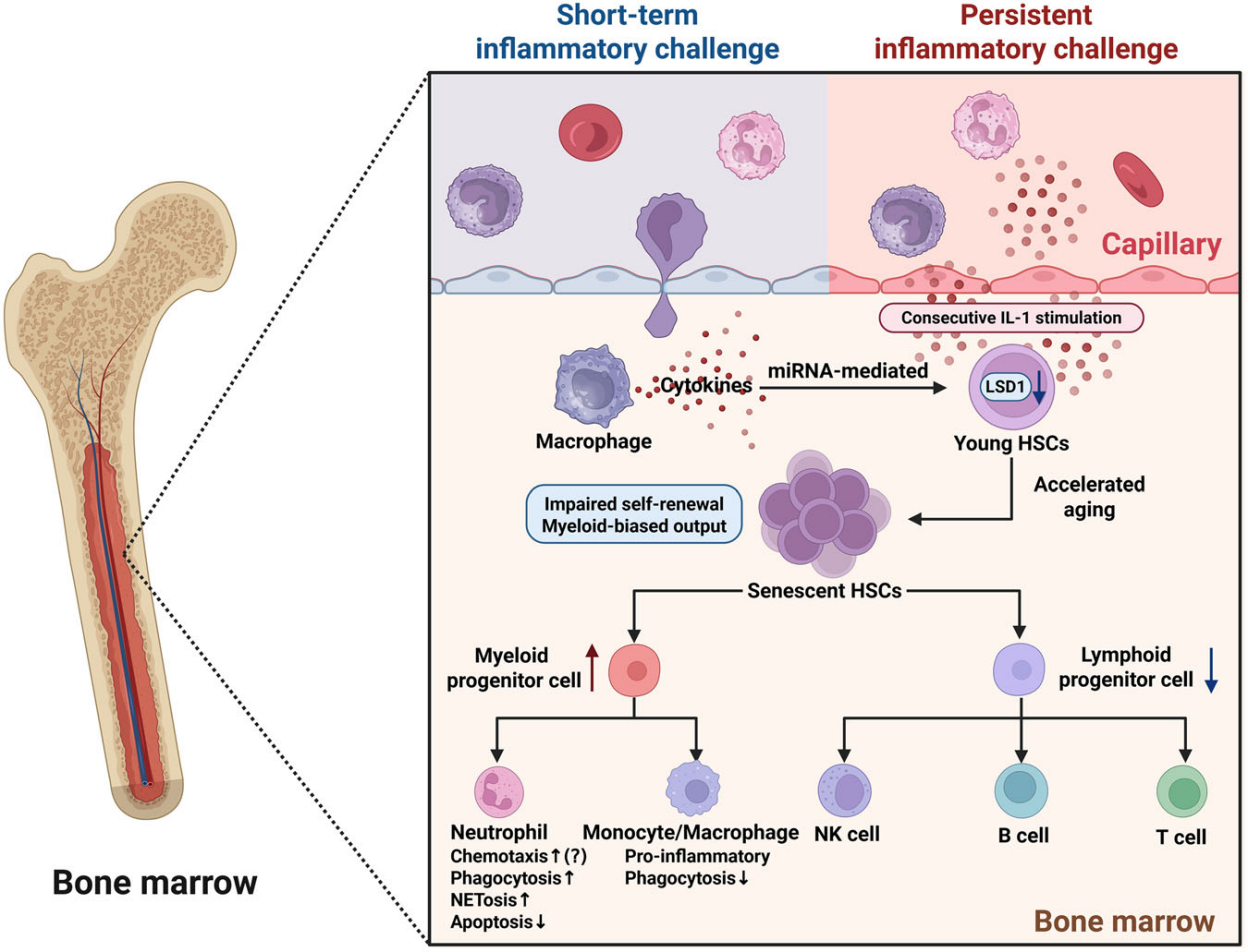
Persistent inflammation or even a short-term inflammatory challenge induces accelerated aging of HSCs. Both transient and persistent inflammatory challenge could induce accelerated aging of HSCs in bone marrow, characterized by impaired self-renewal and myeloid-biased differentiation (increased myeloid progenitor cells and decreased lymphoid progenitor cells). To be specific, during the acute phase of sepsis, overproduction of cytokines from macrophages might induce miRNA-mediated downregulation of LSD1 in HSCs, which causes an acute expansion in hyperinflammatory myeloid progenitors. Additionally, 20 consecutive days of IL-1 stimulation has been demonstrated to induce senescence of HSCs. Then, myeloid progenitor cells could further differentiate into neutrophils and monocytes/macrophages. Aged neutrophils exhibit enhanced chemotaxis, phagocytic activity, and NETs release capacity, along with reduced apoptosis. Of note, the chemotaxis of aged neutrophils remains controversial, with evidence supporting both enhanced and impaired chemotactic capacity. Additionally, aged monocytes/macrophages exhibit a pro-inflammatory phenotype, and the phagocytic activity is suppressed. Decreased lymphoid progenitor cells further differentiate into NK cells, T cells, and B cells. And these cells exhibit a decline in number and/or immune function. *Created with BioRender.com.

However, Milsom et al. have recently suggested that a single inflammatory challenge compromises the functional potency of HSCs and drives accelerated aging of HSCs with no evidence of recovery within one year. Critically, short-term inflammatory stimuli are sufficient to suppress hematopoietic function, which is different from HSCs senescence induced by persistent strong inflammatory stimulation or aging-related chronic low-grade inflammation [11]. Mechanistically, during septic shock, overwhelming release of cytokines might induce miRNA-mediated histone demethylase LSD1 downregulation, which further affects transcription factors with important functions in HSCs [55]. Furthermore, another study has confirmed that emergency hematopoiesis induced by sepsis transiently depletes my-HSCs, while these cells are not only restored, but also increase in number in the bone marrow of sepsis survivors. As a result, sepsis survivors show high levels of neutrophils and monocytes and persistent lymphopenia. Notably, despite the increase in neutrophils and monocytes in bone marrow, these cells exhibit impaired immune capacity and dysregulated maturation, which are correlated with immune dysfunction [56] (Fig. 1).

Taken together, persistent inflammatory stimulation or even a single inflammatory challenge could induce the premature aging of HSCs in young patients with sepsis, leading to excessive inflammation and impaired adaptive immune responses. Moreover, mature immune cells released from bone marrow could be induced to exhibit a prematurely aged phenotype during sepsis, which further promotes disease progression. The effects of immunosenescence on innate and adaptive immunity in young septic patients will be discussed in detail in “Innate immunity and Adaptive immunity”.

### Immunosenescence in elderly patients with sepsis

In elderly patients with sepsis, the synergistic effects of both sepsis and aging may further exacerbate the senescence of HSCs compared with young patients. The sequencing data indicate that after 12 h of LPS stimulation, HSCs from aged mice show stronger inflammatory myeloid-bias differentiation compared with those from young mice due to the upregulation of Klf5, Ikzf1 and Stat3 [53]. Old plasma cells might also accumulate in aging bone marrow and be stimulated to release multiple cytokines via activation of Toll-like receptors (TLRs) signaling, which ultimately results in enhanced myelopoiesis [57]. Additionally, increased permeability of intestinal-barrier in aged individuals can induce the release of various microbial compounds into the circulation, which will drive enhanced IL-1a/b production in the bone marrow. The microbiome/IL-1/IL-1R ultimately contributes to myeloid-bias differentiation in HSCs [30, 58]. In a sepsis model induced by cecal ligation and puncture (CLP), large amounts of gut microbes are released into peritoneum and blood, which might further trigger the aging of HSCs in old mice. Additionally, immune cells derived from HSCs undergo further aging during sepsis, thus immune cells from aged patients exhibit more severe immune dysfunction compared to those from younger patients. This will be discussed in detail in “Innate immunity and Adaptive immunity”.

## Innate immunity

**Fig. 2:**
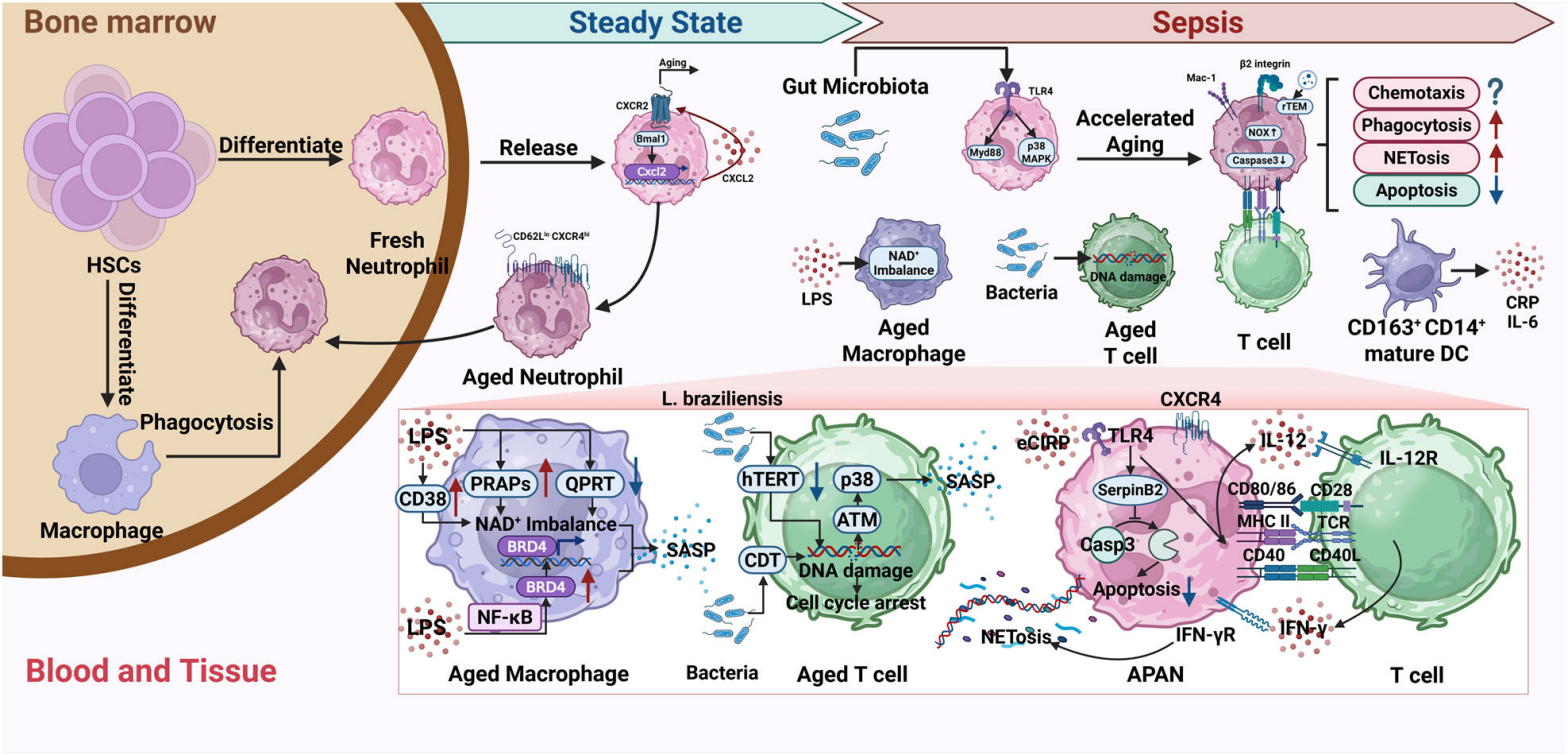
Sepsis induces aberrant aging of immune cells which further exacerbates excessive inflammation. Under physiological conditions, fresh neutrophils are released from bone marrow, and the gene Bmal1 regulates CXCR2-dependent diurnal aging. Then, senescent neutrophils with high CXCR4 and low CD62L expression return to bone marrow and are engulfed by macrophages. However, during sepsis, gut microbiota can induce accelerated aging of neutrophils via TLRs-mediated Mdy88 pathways. And aged neutrophils exhibit functional alterations accordingly. Firstly, these cells are equipped with enhance chemotactic capacity due to the higher expression levels of β integrin via p38 MAPK activation. However, neutrophils with senescent phenotype are also reported to exhibit inaccurate migration, namely rTEM, which is induced by endothelial cell-derived EVs. Therefore, the chemotactic capacity of aged neutrophils needs further investigation. Secondly, aged neutrophils exhibit a higher phagocytic potential due to the enhanced expression levels of phagocytosis receptor Mac-1. Thirdly, they show greater capacity of releasing NETs via activation of NOX. Moreover, eCIRP, DAMPs released during sepsis, can induce APANs (CXCR4^+^CD62L^–^CD40^+^CD86^+^MHCII^+^). This subgroup of neutrophils can release high levels of IL-12 and present antigen to CD4^+^ T cells, which further induce Th1 polarization and the release of IFN-γ. IFN-γ released from T cells could ultimately induced NETosis (shown in the red zoomed-in section). Additionally, eCIRP upregulates SerpinB2 by combining with TLR4 on neutrophils and further suppresses caspase-3-dependent apoptosis. LPS is reported to induce premature aging of macrophages via the NAD^+^ imbalance. As shown in the red zoomed-in section, LPS can upregulate PRAPs and CD38 (increase NAD^+^ consumption), and downregulate QPRT (limit NAD^+^ synthesis), both of which contribute to NAD^+^ imbalance and induce macrophages to exhibit SASP. Moreover, LPS upregulates BRD4 via activation of NF-κB in macrophages, and enhances the release of inflammatory factors. Bacteria are reported to induce premature aging of T cells via DNA damage. As shown in the red zoomed-in section, CDT from bacteria can induce SASP in T cells via DNA damage, since DNA damage will induce cell cycle arrest and promote release of inflammatory cytokines via ATM-p38 axis. Moreover, cutaneous L. braziliensis can downregulate hTERT and impair DNA structure, which might also induce SASP in T cells. Mature CD163^+^CD14^+^ DCs increase in sepsis patients and might contribute to the excessive release of C-reactive protein (CRP) and IL-6. *Created with BioRender.com.

**Fig. 3:**
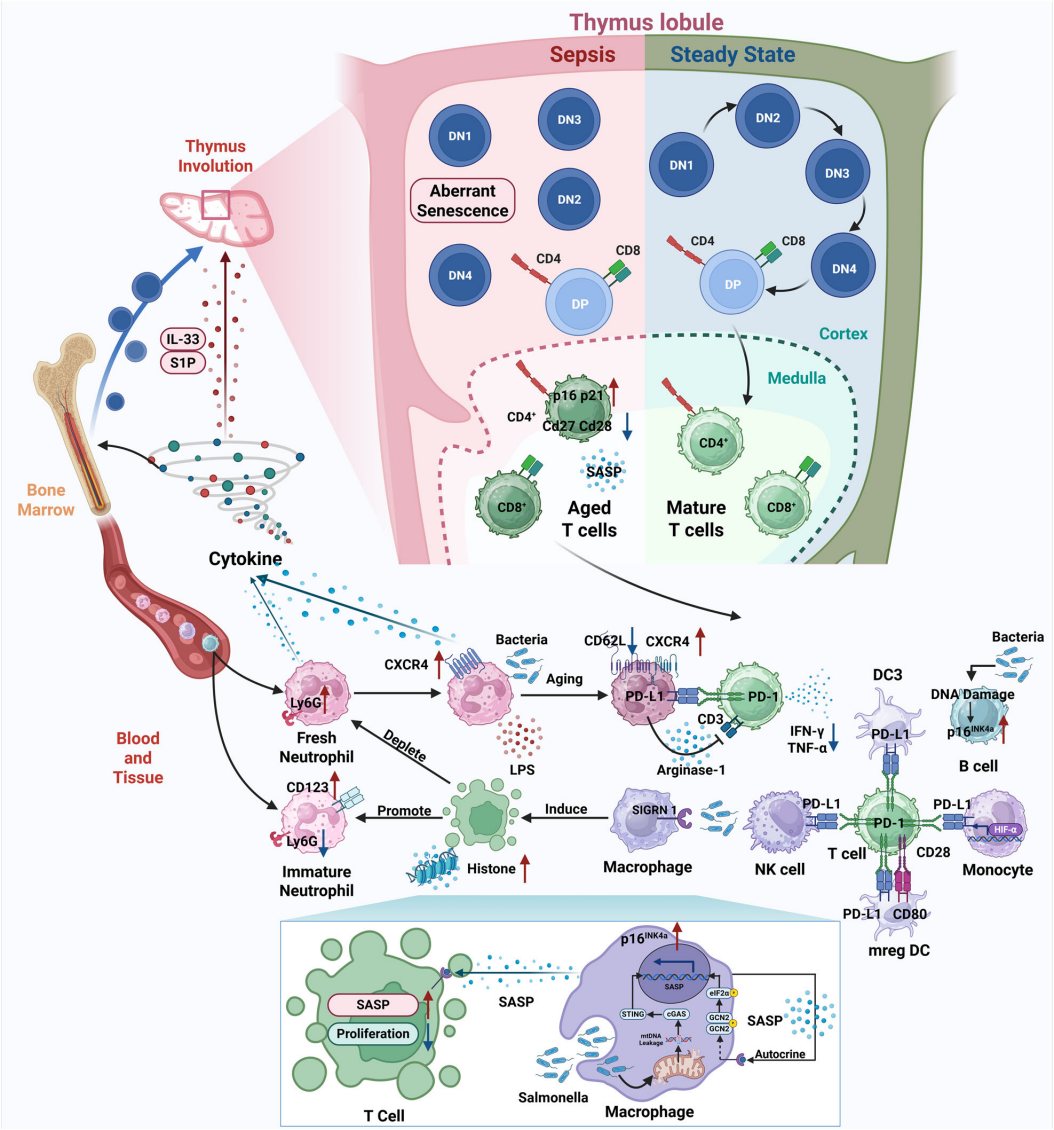
Prematurely aged immune cells during sepsis contribute to immunosuppressive state. After a sustained hyperinflammatory response, in addition to fresh neutrophils (Ly6G^hi^), many immature neutrophils (Ly6G^lo^CD123^hi^) with impaired immune functions are also released into the peripheral blood or tissues. Moreover, microbial capture via phagocytic receptor SIGNR 1 on macrophages induces T cell death-dependent histone release. Cytokines induced by histones will selectively deplete mature neutrophils and increase immature neutrophils. Additionally, bacteria-induced aged neutrophils (CXCR4^hi^CD62L^lo^) inhibit TNF-α and IFN-γ secretion from T cell via upregulation of PD-L1 and the release of arginse-1. Monocytes, NK cells, more mature DC3s and mergDCs also overexpress PD-L1, and further suppress T cell proliferation. However, mregDCs might be also equipped with antigen-presenting ability due to upregulation of CD80. As shown in the blue zoomed-in section, typhoid toxin from Salmonella induces the upregulation of senescent-related gene p16^INK4a^. Macrophages exhibit SASP via activation of cGAS-STING signaling pathway, and macrophages-released SASP-related components can trigger T cell to exhibit SASP and inhibit T cell proliferation. Moreover, SASP-related components could further promote SASP in macrophages via phosphorylation of GCN2 and eIF2α. Additionally, inflammatory cytokine storm during sepsis, including IL-33 or S1P, could directly induce thymus involution or atrophy, which results in aberrant aging of T cells and shows adverse impacts on immune surveillance. To be specific, senescent-related genes, such as p16 and p21, are upregulated, while activation and proliferation-related genes, such as Cd27 and Cd28, are downregulated in CD4^+^ T cells. Bacteria can increase the expression level of p16^INK4a^ in B cells via DNA damage. *Created with BioRender.com.

**Fig. 4:**
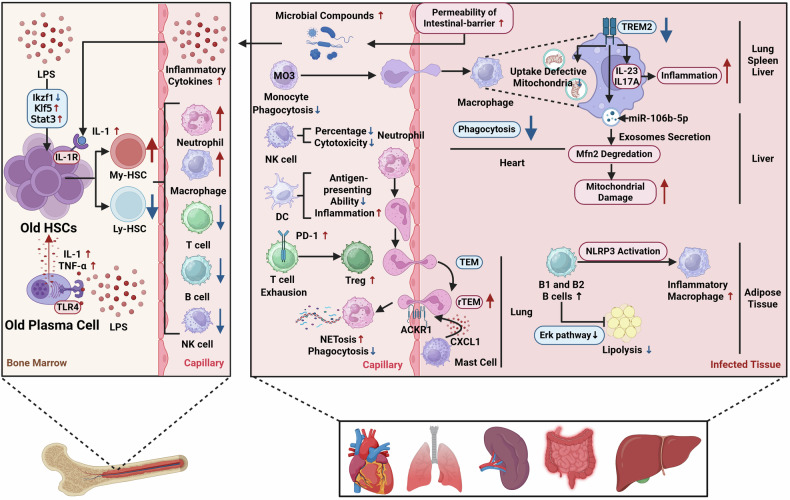
The distinct phenotypic and functional alterations in immune system in aged individuals with sepsis compared with young patients. Increased permeability of intestinal-barrier in elderly patients exacerbates the release of microbial compounds and inflammatory cytokines into the circulation. IL-1, one of the cytokines, will further induce the senescence of HSCs via microbiome/IL-1/IL-1R. LPS can stimulate increased release of IL-1 and TNF-α from old plasma cells via activation of TLR-mediated pathways, which will further induce HSCs aging. Moreover, LPS could directly induce HSCs aging by upregulating Klf5 and Stat3, and downregulating Ikzf1. The old HSCs from elderly septic patients exhibit myeloid-biased differentiation more significantly. In the circulation, neutrophils from older patients exhibit enhanced NETosis and deceased phagocytic capacity compared with those from young patients. Moreover, increased percentage of MO3 (CD14^+^ CD16^++^)/monocytes is observed in old patients, and these cells show impaired phagocytosis. The percentage of NK cells decreases in old patients, and the cytotoxicity of these cells is suppressed. DCs from elderly patients exhibit diminished antigen-presenting ability and enhanced proinflammatory activity. Enhanced T cell exhaustion has been observed in elderly patients due to increased percentage of PD-1^+^ T cells, and the immunosuppressive effects of Tregs are also elevated. In inflamed aged lungs, MC-derived CXCL1 drive neutrophil rTEM via ACKR1-CXCL1 axis. TREM2 expression level decreases in macrophages with aging. In several organs, such as lung, spleen, and liver, the downregulation of TREM2 induced more severe inflammation via IL-23/IL-17A axis. In the heart, TREM2 deficiency macrophages show impaired phagocytosis, which causes decreased uptake of defective mitochondria from cardiomyocytes. In the liver, exosomes-containing miR-106b-5p released from TREM2 deficiency macrophages damage hepatocytic mitochondrial structure and function via Mfn2 blockade. Compared with young septic mice, increased inflammatory B1 and B2 cells are observed in adipose tissues in aged mice. And these cells induce inflammatory macrophages via NLRP3 activation, and inhibit lipolysis via Erk pathway suppression. (The red upward arrows mean increased cell numbers or functions in aged septic individuals compared with those in young septic individuals. The blue downward arrows mean decreased cell numbers or functions in aged septic individuals compared with those in young septic individuals.) *Created with BioRender.com.

During sepsis, the innate immunity is programmed to react immediately to defense against invading pathogens, involving various immune cells such as neutrophils, monocytes/macrophages, dendritic cells (DC), and natural killer (NK) cells [59, 60]. These cells are primarily induced to exhibit a senescent and pro-inflammatory phenotype (Fig. 2). However, persistent inflammatory stimulation might also lead to endotoxin tolerance, which could partially alleviate excessive inflammation but may also induce immunosuppression. Additionally, aged innate immune cells could mediate immunosuppression through interactions with lymphocytes (Fig. 3). Elderly sepsis patients exhibit a more pronounced decline in innate immune functions compared to younger patients (Fig. 4).

### Neutrophil

Neutrophils play a critical role in innate immune defense, since they represent the first line of cellular defense against infection [61]. Under physiological conditions, the anti-microbial immunity of neutrophils follows a circadian pattern [62]. Hidalgo et al. elucidate that the gene Bmal1 in neutrophils regulates chemokine CXCL2 expression and further induces chemokine receptor CXCR2-dependent diurnal aging. This diurnal alteration regulates the migration of aging neutrophils out of circulation, thereby inhibiting overwhelming vascular inflammation. This research emphasizes that neutrophil senescence is intrinsically driven, since they failed to find other factors [63]. However, accelerated aging has been reported to be triggered by acute stress or inflammatory responses in recent years [64]. Moreover, neutrophils exhibit impaired immune functions with aging, such as defective chemotaxis and phagocytosis, while prematurely aged neutrophils (CXCR4^hi^CD62L^lo^) during sepsis show enhanced immune functions in certain aspects.

#### Sepsis-related premature aging of neutrophil

In models of septic shock and sickle-cell disease, neutrophil aging is induced by microbiota via TLRs and Myd88-mediated signaling pathways [15]. Previous studies have indicated that aged neutrophils show impaired migration to inflamed tissues and reduced pro-inflammatory activity [65, 66]. However, Reichel et al. have demonstrated that compared with fresh neutrophils, aged neutrophils express higher levels of β2 integrins in high-affinity conformation and therefore are equipped with enhanced capacity of migration via TLR4 and p38 MAPK-dependent pathways [67]. Additionally, these “experienced defenders” exhibit a higher phagocytic potential that is dependent on the phagocytosis receptor Mac-1/CD11b and spleen tyrosine kinase-mediated pathways. And they also show greater capacity of releasing neutrophil extracellular traps (NETs, webs of DNA decorated with anti-microbial proteins) mainly regulated by activated NADPH oxidase (NOX) [15, 67–69]. Furthermore, Wang et al. have proven that extracellular cold-inducible RNA-binding protein (eCIRP), which acts as damage-associated molecular patterns (DAMPs) fueling inflammation during sepsis, can enhance the expression level of SerpinB2 in neutrophils via TLR4 pathway and further suppress caspase-3-dependent apoptosis [70]. Defective apoptosis is considered to induce aged neutrophil with an enhanced capacity to release NETs by prolonging their lifespan [71]. Treatment with recombinant junctional adhesion molecule-C (JAM-C) which inhibits bone marrow-derived neutrophils (BMDN) apoptosis, could increase the frequences of CXCR4^+^ aged neutrophils and further exacerbate sepsis-induced lung injury [72]. Additionally, Wang et al. have also found that eCIRP can induce a special neutrophil subgroup, featured by cell surface markers of antigen-presenting cells (APCs) and senescent neutrophils, and they name this subpopulation as antigen-presenting aged neutrophils (APANs). APANs can release high levels of IL-12 and present antigen to CD4^+^ T cells, which further induce Th1 polarization and IFN-γ-dependent excessive NETosis [64]. Although NETs limit pathogen dissemination effectively, excessive release of NETs from neutrophils will exacerbate inflammatory responses [73, 74]. Additionally, defective homing of aged neutrophils might also contribute to increased proportion of prematurely aged neutrophils in infection sites or peripheral blood. Senescent CXCR4^+^ neutrophils home to bone marrow where express stromal cell-derived factor-1alpha (SDF-1α) [75]. However, during sepsis, granulocyte colony-stimulating factor (G-CSF) increases in bone marrow to induce emergency myelopoiesis [56]. And G-CSF was reported to decrease bone marrow SDF-1α [76, 77], which might hinder the homing of CXCR4^+^ neutrophils to the bone marrow. Notably, to date, the relevant hypotheses have not been validated in the sepsis model. Therefore, the impact of sepsis on neutrophil homing requires further investigation.

Of note, recent research suggests that endothelial cell-derived extracellular vesicles (EVs) induce abnormal neutrophil trafficking, namely reverse transendothelial migration (rTEM) during sepsis, which contributes to inaccurate migration and remote organ injury. Importantly, scRNA-seq data suggest that the rTEM neutrophils belong to a specific subgroup characterized by ICAM1^hi^CXCR4^hi^CXCR1^lo^CD62L^lo^. And this subpopulation might exhibit a senescent phenotype due to the high expression of CXCR4 and low expression of CD62L [78]. The result might argue against conclusions mentioned above, which indicate that aging neutrophils firstly migrate to infective sites. One of the possible reasons is subset heterogeneity. Aged neutrophils could be further subclassified in different subtypes due to the wide application of scRNA-seq. Gut microbiota-induced aged neutrophils might be equipped with enhanced chemotactic capacity, while EVs-induced aged neutrophils exhibit features of rTEM. Another contributing factor may be disease stage dependency. Since the occurrence of rTEM is primarily driven by interactions between damaged endothelium and neutrophils, prematurely aged neutrophils in severe sepsis tend to exhibit impaired chemotactic capacity.

Aside from exacerbating inflammation, aged neutrophils could also induce the shift towards immunosuppression during sepsis. Aged neutrophils exhibit increased expression levels of PD-L1 and arginase-1 after LPS stimulation, which may show enhanced inhibition of TNF-α and IFN-γ secretion from CD8^+^ T cells [79]. Moreover, NETs released from neutrophils enhance cholesterol metabolism in naïve CD4^+^ T cell, which induces them to differentiate to Tregs and further results in immunosuppressive state [80]. Notably, persistent inflammatory responses induced by aging immune cells may trigger massive immature neutrophils to recruit from bone marrow into circulation [81]. A clinical trial has confirmed that circulating immature granulocytes are linked with early sepsis deterioration and are responsible for immunosuppression through killing activated T cells [82]. Another clinical trial also indicates that increased blood levels of immature neutrophils may be associated with poor prognosis [83]. Moreover, unsupervised analysis of high-dimensional mass cytometry data suggest that CD123^+^ immature neutrophils are correlated with clinical severity of sepsis [84]. Papayannopoulos et al. have also demonstrated that microbial capture via phagocytic receptor SIGNR 1 on macrophages induces T cell death-dependent histone release. Cytokines induced by histones will selectively deplete mature Ly6G^high^ neutrophils and increase immature Ly6G^low^ neutrophils with a defective potential oxidative burst, which may ultimately damage innate and adaptive immunity [85].

In summary, sepsis can induce premature aging of neutrophils, leading to phenotypic and functional changes that exacerbate inflammatory responses. Moreover, the increased proportion of aged neutrophils may further contribute to immunosuppression by interacting with adaptive immune cells and inducing the release of immature neutrophils.

#### Neutrophils in aged individuals with sepsis

The aging of both hosts and cells is closely linked, and host aging results in specific alterations in neutrophil phenotype and function [86]. Notably, neutrophils in elderly sepsis patients show some differences from prematurely aged neutrophils from young patients. First of all, neutrophils from old patients exhibit preserved chemokinesis, namely random migration, while chemotaxis is impaired. Therefore, these aged neutrophils can not accurately migrate towards inflammatory sites [87–89]. To be specific, there are no differences in surface expression of chemokine receptors with age. However, neutrophils from old subjects exhibit weak migratory accuracy via enhanced PI3K activation. Moreover, these aged cells are equipped with increased degranulation and proteinase activity, which may further aggravate multiple organ damage [90]. Nourshargh et al. have demonstrated that mast cells (MC) and MC-derived CXCL1 drive neutrophil rTEM in inflamed aged tissues via ACKR1-CXCL1 axis, which exacerbates remote organ damage [91]. Combined with the conclusion presented in “Sepsis-related premature aging of neutrophil”, it is likely due to the increased proportion of rTEM aging neutrophil subset in elderly septic group. Additionally, neutrophils from elderly people are reported to generate more reactive species in closer proximity to endothelial cells due to elevated expression level of adhesion molecule CD11b, which may contribute to damaged endothelial barrier [92]. Aside from inaccurate migration, senescent neutrophils exhibit impaired bactericidal capacity. For example, the phagocytosis of aged neutrophils is weakened due to the imbalance of FcγR and CD11b expression [93]. Moreover, these cells also show abnormal TLR function and increased apoptosis [38, 94, 95]. Notably, previous literature has suggested that the ability of neutrophils to form NETs is impaired in the elderly [94, 96]. However, recent scRNA-seq data indicate that the NET formation pathway is significantly enhanced in old septic patients compared with that in young patients [17]. The possible explanation for this discrepancy is that earlier studies were primarily conducted in vitro due to methodological limitations, which may not fully recapitulate the in vivo dynamics of neutrophil function. In contrast, recent studies have employed high-resolution analytical techniques in clinical samples and animal models, providing a more comprehensive understanding of NET formation in aging neutrophils under septic conditions.

### Monocyte/macrophage

Monocytes and macrophages play a critical role in inflammatory processes during sepsis [97]. Activated macrophages are typically classified into two distinct categories: pro-inflammatory M1-like macrophages and anti-inflammatory M2-like macrophages [98]. Senescent monocytes/macrophages show downregulated TLR expression and impaired immune functions, such as decreased chemotaxis, phagocytosis and antigen-presenting capacity [10, 99, 100]. And a study has suggested that breakdown of NAD^+^ synthesis might underline the defective innate immune responses in aged macrophages [101].

#### Sepsis-related premature aging of monocyte/macrophage

Andreasson et al. have demonstrated that after the stimulation of LPS, macrophages increase NAD^+^ consumption by PARPs, and decline QPRT expression that limits de novo NAD^+^ synthesis [101]. Imbalance in NAD^+^ homeostasis is closely correlated with aging and a variety of diseases, and NAD^+^ has been found to exhibit anti-inflammatory properties [102]. Therefore, NAD^+^ supplement by replenishing QPRT could induce macrophages from a pro-inflammatory phenotype with reduced phagocytic potential, which is similar to an aging state, to an anti-inflammatory and phagocytosis-competent state [101, 103]. Another study also indicates that macrophage inflammation could be alleviated by promoting RNF146-dependent PARP1 ubiquitination [104]. Furthermore, after the treatment of LPS, macrophages show a higher expression level of CD38 in vivo and in vitro [33, 105]. Since NADase CD38 plays a critical role in age-related NAD^+^ decrease, CD38^+^ macrophages exhibit a senescent and pro-inflammatory phenotype [103, 106]. Moreover, LPS has been reported to induce macrophage aging with high expression level of BRD4, a protein that regulates the transcription of genes involved in inflammation [107], apoptosis [108] and cell cycle progression [109], through the activation of NF-κB signaling pathway [110].

Notably, long-term exposure of LPS can induce an endotoxin tolerance state with a reprogrammed inflammatory response, namely an inactivation of monocytes and macrophages [111]. Wang et al. have suggested that macrophage M2 polarization is correlated with endotoxin tolerance [112]. The alteration in immune responses could alleviate excessive inflammation, while it might also induce immunosuppression during sepsis [113, 114]. During endotoxin tolerance, monocytes have been reported to overexpress PD-L1 driven by translocation of hypoxia-inducible factor-1α (HIF1α) into the nucleus [115]. These results might indicate that monocytes are induced to exhibit a senescent phenotype and further inhibit T cell proliferation, since senescent cells are recognized to upregulate PD-L1 and modulate T cell responses [116, 117].

Overall, sepsis induces macrophage to exhibit an aged and pro-inflammatory phenotype. However, long-term stimulation of LPS might result in inactivation of monocytes/macrophages, which would ultimately diminish immune functions.

#### Monocytes/macrophages in aged individuals with sepsis

During sepsis, although the number of macrophages seem preserve in the elderly, these cells exhibit age-related functional impairment, such as suppressed chemotaxis, phagocytosis and antibacterial effects [118]. For example, Fan et al. have proven that macrophages isolated from the elderly group show reduced filamentous actin polymerization signaling pathway, which further contributes to impaired phagocytosis, due to reduced Rac1 expression level [119]. Additionally, TREM2 expression level has been reported to be decreased in both elderly septic patients and aged septic mouse models, which will further result in more severe inflammation via the upregulation of IL-23/IL-17A axis [120]. Fang et al. have suggested that TREM2 deficiency macrophages show impaired capacity of self-renewal, which further causes defective clearance of damaged mitochondria from cardiomyocytes and overwhelming inflammatory responses during sepsis [121]. Moreover, this research team also proves that exosomes-containing miR-106b-5p released from TREM2 deficiency macrophages damage hepatocytic mitochondrial structure and function via Mitofusin 2 (Mfn2) blockade [122]. Therefore, multiple studies have emphasized that targeting TREM2 might be a promising therapeutic strategy during sepsis [123–126].

LeVan et al. have found that circulating leukocytes, such as monocytes, release elevated levels of TNF-α with aging. However, increased production of TNF-α is not parallel with TLR2 and TLR4 expression levels on leukocytes, which may cause increased susceptibility to sepsis in the elderly [127]. A clinical trial indicates that the expression level of tissue factor (TF) on classical monocytes (MO1, CD14^++^CD16^-^) is lower in older sepsis patients, which is correlated with poorer prognosis [128]. Moreover, another clinical trial has suggested that the percentage of MO3 (CD14^+^ CD16^++^)/monocytes increases in old septic patients, and it is also closely linked to prognosis [129]. These “non-classical” monocytes are equipped with stronger capacity to release pro-inflammatory factors [130]. However, these cells exhibit impaired phagocytic capacity due to the low expression levels of chemokine receptors [129]. This implies that the amplification of this monocyte subset in older patients may ultimately cause excessive inflammation and pathogen dissemination. The result of another study indicates that the increased level of platelet–monocyte aggregate (PMA) is correlated with the mortality in elderly septic patients. While there is no significant correlation between PMA and the mortality in younger patients [131]. Additionally, Meng et al. have demonstrated that old mice exhibit more severe inflammation and cardiac functional suppression due to elevated level of monocyte chemoattractant protein-1 (MCP-1) [132].

Taken together, monocytes/macrophages in the elderly with sepsis show impaired immune efficacy compared with those in young patients. And this might further exacerbate inflammatory responses and immunosuppression.

### DC

DCs are the most effective APCs, bridging innate and adaptive immunity [133]. In aged subjects, there is no significant changes in the number and phenotype of DC [10]. However, DCs from the elderly individuals primarily exhibit defective antigen-presenting capacity.

#### Sepsis-related premature aging of DC

A study has identified a subset of “mature DCs enriched in immunoregulatory molecules” (mregDC) in septic models. Specifically, this DC subset is at the terminal stage of differentiation, and it could effectively induce the phenotypic shift of naïve T cells towards Tregs [134]. Additionally, another research team has also observed a shift towards a more mature CD163^+^CD14^+^ phenotype within DC3 subgroup in COVID-19 patients, which is associated with inflammatory markers and disease severity. Moreover, DC3 subset shows upregulated expression level of PD-L1 and impaired capacity to activate T cells [135]. It is worth noting that there are some differences between a more mature DC phenotype and a senescent DC phenotype, and further researches in this area are needed.

#### DCs in aged individuals with sepsis

Compared with young individuals, DCs isolated from old people exhibit impaired immune functions, such as defective phagocytosis and excessive release of inflammatory mediators, which may result from suppressed phosphorylation of AKT [136]. A study has indicated that OT-I CD8^+^ T cell expansion is impaired in aged mice during infection, which further selectively influences CD8α^+^ DC. Specifically, CD8α^+^ DC subset shows a decreased expression levels of costimulatory molecules. And the reduction in CD8α^+^ DCs ultimately suppresses CD8^+^ T cell accumulation [137]. Notably, DCs from old septic patients show suppressed antigen-presenting ability and turn into a pro-inflammatory and senescent phenotype compared with those in young sepsis patients [17].

### NK cell

NK cells are lymphocytes of the innate immunity that mediate important effector functions in control of malignancy and viral infection [138]. NK cells undergo a developmental transition from newborns to adults, marked by the upregulation of the Killer-cell Immunoglobulin-like Receptor (KIR) family and a concurrent downregulation of inhibitory Natural Killer Group 2 Member A (NKG2A) receptors. This phenotypic shift remains stable in the majority of elderly individuals. However, a minority of elderly individuals exhibit a notable downregulation of cytotoxicity-activating receptors, which might cause impaired innate immune functions [10].

#### Sepsis-related premature aging of NK cell

At present, there is few research on sepsis-related NK cell aging in young septic patients. An observational study has suggested that increased percentage of PD-L1^+^ NK cells is associated with poor prognosis in sepsis patients, and these cells might play a vital role in immunosuppression during sepsis [139].

#### NK cells in aged individuals with sepsis

Several studies have suggested that aging causes an increase in the number of NK cell in the elderly [38]. However, according to the results of observational studies, the proportions of NK cells in aged septic individuals show a significant decrease compared with those in young patients [140]. And there is a negative correlation between the percentage of NK cells in lymphocytes and 28 day mortality in sepsis [141]. Functionally, NK cells in elderly individuals show normal or increased release of IFN-γ, but their cytotoxicity is suppressed. This functional alteration may contribute to the aggravated tissue damage in the elderly [94].

## Adaptive immunity

One of the hallmarks of immunosenescence is impaired adaptive immunity. Sepsis-induced aged lymphocytes, including T cells and B cells, primarily exhibit an exhausted-like/immunoregulatory phenotype, which further contributes to increased susceptibility to infections. Additionally, SASP in lymphocytes might also lead to increased levels of cytokines and prolonged inflammatory responses. Notably, elderly people with sepsis show a more pronounced impairment in adaptive immunity compared with younger patients.

### T cell

T cells serve as the major effector cells in cellular immunity. After activation, these cells are mainly categorized into two functionally distinct subgroups: CD4^+^ T helper cells and CD8^+^ cytotoxic T cells. The effective activation, proliferation, clonal expansion, and effector function of both CD4^+^ and CD8^+^ T cells are essential for the efficient clearance of infections caused by pathogens [142]. A reduction in the population of circulating naive CD8^+^ T cells is the most prominent and consistently observed indicator of immunosenescence in healthy elderly individuals. Additionally, the proportion of memory T cells and Tregs increases in elderly people. The decreased T cell receptor diversity could further result in defective responsiveness to neoantigens [38].

#### Sepsis-related premature aging of T cell

Several studies have proven that T cell aging is accelerated in some infectious diseases, thereby exacerbating systemic inflammation. Krejsgaard et al. have confirmed that bacterial genotoxins, specifically cytolethal distending toxin (CDT), can induce SASP in activated T cells via DNA damage. And the SASP is orchestrated by the ATM-p38 axis which further promotes persistent inflammation due to the increased release of TNF-α, various interleukins and chemokines [143]. Additionally, Gomes et al. described the accumulation of circulating T cells showing telomere dependent-aging, such as short telomeres and low expression level of hTERT, during cutaneous L. braziliensis infection. This subgroup of T cells exhibits a pro-inflammatory phenotype and is strongly linked with systemic inflammation [144].

Of note, aged T cells primarily result in the impairment of adaptive immunity and the shift towards an immunosuppressive state during sepsis. Zeng et al. have demonstrated that early T lineage progenitors (ETPs) show a dramatic decrease and impaired migration to thymus in septic models, which result in reduced lymphopoiesis [145]. Furthermore, sepsis-induced thymus involution or atrophy is closely associated with T cell aging and defective adaptive immune responses. Chen et al. have reported that IL-33 induced by severe infection causes aberrant aging in naïve T cell through thymic involution [146]. Knethen et al. have also proven that decreased apoptosis of immature single and double positive thymocyte during sepsis results in thymus involution [147]. Moreover, in septic patients, Hecker et al. have found reduced thymic output and enhanced aging of lymphocytes that ultimately cause lymphopenia [148]. Another study also indicates that septic mouse models exhibit thymus atrophy via increased level of sphingosine-1-phosphate (S1P), which diminishes capacity to fight against secondary infections [149, 150]. Aging T cell exhibits reduced TCR-dependent proliferation and increased levels of SASP-related cytokines, both of which can result in immune dysfunction [151]. Additionally, recent studies have demonstrated that typhoid toxin can induce mitochondrial dysfunction in macrophages, and the mtDNA released from damaged mitochondria will initiate SASP-related cytokines production, such as TNF-α, IL-8, IL-18, via activation of cGAS-STING signaling pathway. SASP-related components can trigger the aging of CD4^+^ and CD8 T^+^ cells that may have adverse impacts on immune surveillance [14, 152].

In conclusion, accelerated aging of T cells has been observed during sepsis. On the one hand, the increased release of SASP-related cytokines could aggravate inflammatory responses. On the other hand, these aged cells severely compromise adaptive immune function.

#### T cells in aged individuals with sepsis

T cell exhaustion has been found in elderly septic models, which is characterized by the reduction of effector T cells and the increase of PD-1^+^CD8^+^ T cells [153, 154]. Moreover, the immunosuppressive effects of Tregs are reported to be enhanced in old septic patients compared with young patients [17]. The results of a clinical research indicate that high levels of soluble urokinase plasminogen activator receptor (suPAR) in elderly septic patients may contribute to the imbalance of Th1/Th2 cells. And it further results in impaired immune responses to infection [155]. Another study also suggests that aging is associated with decreased IL-23 responsiveness of CD8^+^ memory T cells, defective capacity of inducing CD4^+^/IL-23r, and reduced Th-17 responsiveness. And these alterations could ultimately cause poorer prognosis in the elderly [156].

### B cell

B cells, as the unique source of diverse immunoglobulin repertoires, constitute an essential component of humoral immunity [157]. These cells exert their immune functions through antigen presentation, secreting cytokines, and producing antibodies [158]. In older adults, there is a decline in naïve B cell populations, accompanied by an increased proportion of memory B cells. Notably, the release of antibodies decreases, which will result in increased susceptibility to infections in elderly individuals [38].

#### Sepsis-related premature aging of B cell

Chronic inflammation has been reported to induce premature B cell aging [159]. Another in vitro experiment has demonstrated that LPS treatment could promote B cell over-proliferation, which in turn induces p16^INK4a^ expression and cellular aging through DNA damage. [160]. However, Venet et al. have found that the circulating B cells from septic shock patients exhibit a CD21^low^CD95^high^ exhausted-like phenotype, while their maturation status remains unchanged [161]. Moreover, septic patients with poorer prognosis show impaired B cell maturation [162]. Therefore, premature B cell aging induced by sepsis needs further studies.

#### B cells in aged individuals with sepsis

Adipocyte lipolysis is reported to protect mice against infection [163]. Carey et al. observe an accumulation of inflammatory B1 and B2 B cells in visceral white adipose tissue (vWAT) from aged mice. And these types of B cells further induce inflammatory macrophages via NLRP3 activation, and inhibit non-canonical vWAT lipolysis through suppression of Erk signaling pathway [164]. This will ultimately result in a persistent inflammatory state. Additionally, a clinical study indicates that elderly septic patients exhibit decreased naïve B cells and enhanced B cell exhaustion. Insufficient production of IgM and increased CD21^+^ exhausted B cells will ultimately contribute to immunosuppression and secondary infections [165].

## Potential therapeutic strategies targeting immunosenescence

**Fig. 5:**
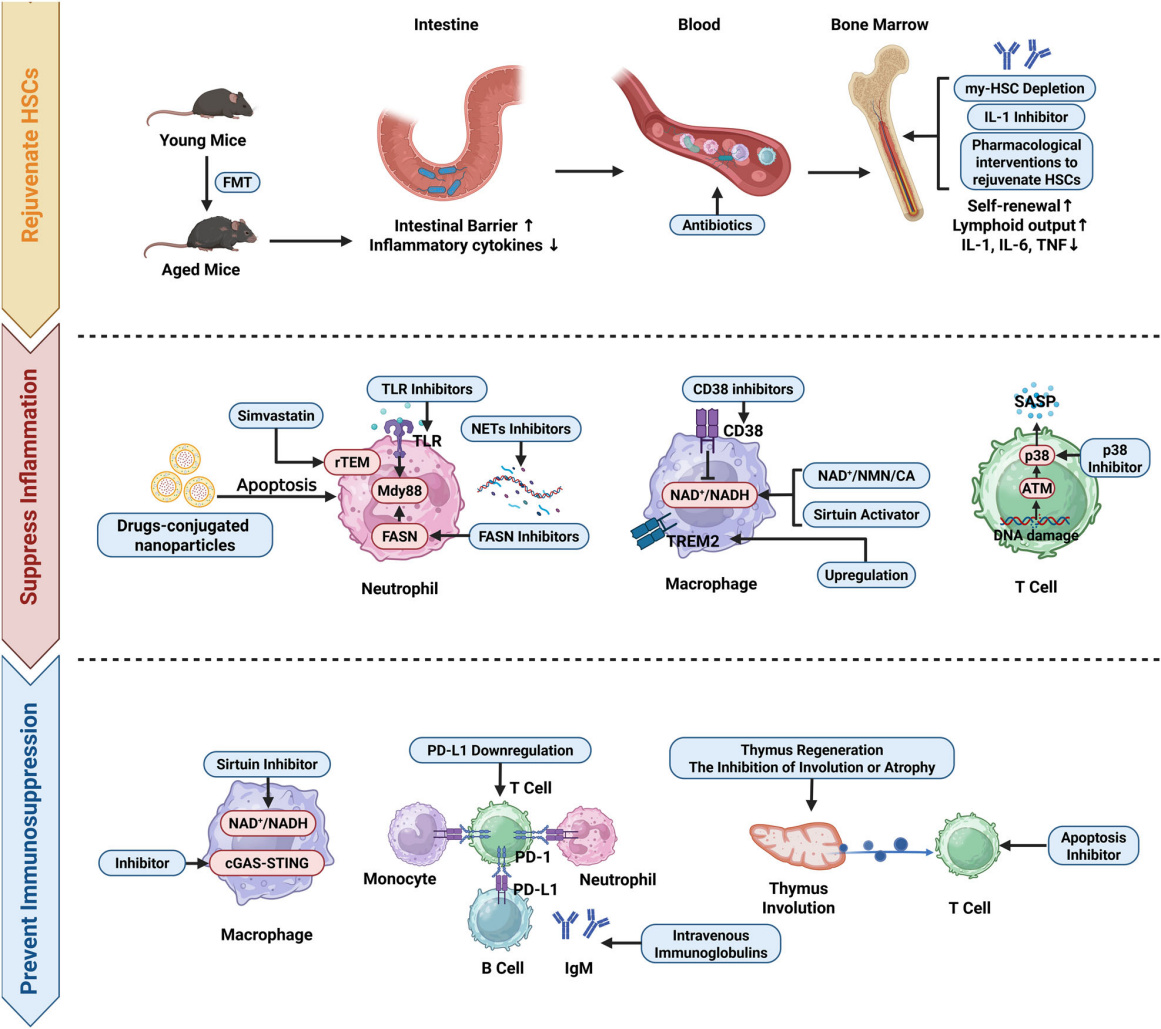
Potential therapeutic strategies targeting immunosenescence. The potential treatments targeting immunosenescence could be classified as three aspects. Firstly, rejuvenating HSCs is a promising therapeutic strategy. FMT from young mice could enhance intestinal barrier and further reduce inflammatory cytokines in aged mice. Antibiotic treatment can also effectively eliminate bacteria in the circulation. Moreover, my-HSC depletion, IL-1 inhibitors and pharmacological interventions, could promote HSCs self-renewal, increase lymphoid output and suppress the release of inflammatory cytokines. Secondly, during early phase of sepsis, treatments targeting immunosenescence might suppress excessive inflammation. It is recommended to use TLR inhibitors or FASN inhibitors, which suppress TLRs/Mdy88-mediated signaling pathways, in order to inhibit accelerated aging of neutrophils. Nanoparticles-containing drugs could selectively promote neutrophil apoptosis and prevent the persistent existence of aged neutrophils. High-dose simvastatin could enhance the accuracy of neutrophil migration and thereby prevent remote organ damage. Since aged neutrophils exhibit an enhanced capacity to release NETs, NETs inhibitors can be used to suppress inflammation. Moreover, restoring the balance between NAD^+^ and NADH by using CD38 inhibitors, NAD^+^/NMN/CA, and Sirtuin activators, could induce macrophages to exhibit an anti-inflammatory phenotype. The upregulation of TREM2 could also suppress the senescent pro-inflammatory phenotype of macrophages. The usage of p38 inhibitor might inhibit SASP in T cells. Thirdly, it is necessary to prevent immunosuppressive state in the late stage of sepsis. Sirtuin inhibitors or the suppression of cGAS-STING signaling pathway might restore immune functions of macrophages. Moreover, the downregulation of PD-L1 on neutrophils, monocytes or B cells might reduce their interactions with T cells, which could inhibit T cell exhaustion and restore adaptive immune functions. The usage of IgM-enriched intravenous immunoglobulins and apoptosis inhibitors targeting lymphocytes might also improve the prognosis of patients. Finally, thymus regeneration or inhibiting the involution or atrophy of thymus can be tested in septic models or patients in the future. *Created with BioRender.com.

Since the impacts of immunosenescence cross the entire period of sepsis, we propose three therapeutic strategies targeting immunosenescence to improve prognosis, including rejuvenating HSCs in bone marrow, suppressing excessive inflammation induced by aged immune cells, and preventing immunosenescence-mediated immunosuppression (Fig. 5, Table 1).

**Table 1:** Potential therapeutic strategies targeting immunosenescence.

The hallmarks of immunosenescence	Potential targets	Therapies	Effects	Ref
Increased my-HSCs	Rejuvenate aged HSCs	The depletion of my-HSCs	Depletion of my-HSCs attenuates senescent phenotypes, reduces inflammatory markers and enhances adaptive immunity.	[29]
		FMT from young mice	Enhance lymphoid differentiation, reduce myeloid differentiation and suppress inflammatory signals.	[168]
		Inhibit microbiota using antibiotics	Inhibit my-HSCs and alleviate inflammation. However, the timing and duration of antibiotic therapy need further researches.	[30, 174, 175]
		The inhibition of inflammatory cytokines, especially IL-1	Exhibit alleviated aging-related inflammatory signatures.	[30, 176–178]
		Pharmacological interventions	Rapamycin, CASIN, TN13, SB203580 and ABT263 increase the regenerative capacity of HSCs and enhance immune responses.	[181]
Excessive inflammation induced by senescent immune cells	Reduce the quantity of aged neutrophils and enhance their functions	Prevent accelerated aging induced by TLRs-mediated pathways	The usage of TLR4 inhibitors, such as TAK-242, C34 or E5564, and the treatment of FASN inhibitor, C75, might attenuate aberrant aging and mitigate aggravated inflammation.	[184–188]
		Promote the apoptosis of aged neutrophils	The drugs-conjugated nanoparticles selectively promote neutrophil apoptosis and might attenuate persistent inflammation. Moreover, the downregulation of PD-L1 might also mitigate inflammation.	[199–201]
		Suppress rTEM	CXCL1 blockade or high-dose simvastatin enhances the accuracy of neutrophil migration.	[88, 91]
	Induce an anti-inflammatory phenotype of macrophages	Increase the ratio of NAD^+^/NADH	The administration of NAD^+^, NMN, CA, NADase CD38 blockers or Sirtuin activators could induce the shift from a senescent pro-inflammatory phenotype to an anti-inflammatory phenotype.	[214–222]
		The upregulation of TREM2	Triggering TREM-2 could enhance bacterial clearance and protect against inflammatory lesions.	[120, 121, 126]
	Suppress the SASP in T cells	Block p38 MAPK signaling pathway	Since ATM-p38 axis plays a critical role in genotoxin-induced SASP in T cells, the usage of p38 MAPK inhibitors, such as BIRB 796, might inhibit persistent inflammation.	[143, 228, 229]
Immunosuppression mediated by aged immune cells	Suppress the interactions between T cells and aged innate immune cells	The downregulation of PD-L1 expression level or the inhibition of NET release in neutrophils	Hamper the interactions between neutrophils and T cells, which further prevents T cell exhaustion and the differentiation to Tregs.	[79, 80]
		Enhance the anti-bacterial capacity of macrophages	The inhibition of Sirtuins restores bacterial phagocytosis of macrophages.	[232–234]
	Improve the proliferation and immune function of aged T/B cells	Delay the senescence of thymus	The inhibition of thymus involution or atrophy could promote lymphocytosis. And thymus regeneration or IL-7 administration might be a promising therapy in the future.	[149, 249, 250]
		Pharmacological interventions	The usage of DcR3, baicalin, anti-LAG-3 antibody could suppress lymphocyte apoptosis or promote proliferation.	[241–243]
		IgM-enriched intravenous immunoglobulins or trimodulin	The intravenous immunoglobulins might help to improve adaptive immunity.	[165, 245, 247]

### Rejuvenate HSCs in bone marrow

Since increased proportion of my-HSCs in the old septic patients can induce excessive inflammation and defective adaptive immune responses, Weissman et al. have suggested that depletion of my-HSCs rejuvenates aged immunity, reduces inflammatory markers and enhances adaptive immunity [29]. Additionally, microbiota signals are reported to promote the expansion of my-HSCs at the expense of lymphopoiesis [166, 167]. Therefore, Qian et al. indicate that fecal microbiota transplantation (FMT) from young mice rejuvenates aged HSCs in old mice, including enhanced lymphoid differentiation, reduced myeloid differentiation and suppressed inflammatory signals [168]. Additionally, the suppression of microbiota through antibiotic treatment has been reported to reverse my-HSCs in older mice, which may contribute to the alleviation of inflammatory responses [30]. Although the usage of antibiotics has been a standard treatment in sepsis [169], a clinical trial proves that the usage of broad-spectrum antibiotics results in gut microbiota disruption in healthy young man, who are administrated LPS to induce a transient sepsis-like syndrome. However, the antibiotic-induced gut microbiota disruption does not further affect innate immune responses in these healthy subjects [170]. More researches are required to validate this hypothesis in sepsis patients, since these patients have already exhibited significant alterations in their gut microbiota, and the usage of antibiotics might exacerbate this disruption and further impair immune functions [171]. Additionally, the inappropriate use of antibiotics may cause drug resistance and show no significant improvement in many sepsis patients [172, 173]. Therefore, the timing and duration of antibiotic therapy need further researches [174, 175]. Exploring the mechanisms underlying microbiota signal-induced HSC aging might lead to more rational use of antibiotics in treatment. Moreover, several studies have demonstrated that the exposure of inflammatory environment, especially IL-1, could mediate HSCs senescence [30, 176]. Therefore, inhibition of IL-1 signaling can be an effective therapy [177, 178]. In clinical trials, treatments targeting IL-1 signaling have shown promising efficacy in patients with sepsis. For example, anakinra, an IL-1α/β inhibitor, has been shown to reduce 28 day mortality and hospital stay [177, 179]. Another clinical trial also demonstrated that the treatment of recombinant human interleukin-1 receptor antagonist (rhIL1RA) could reduce 12% 28 day mortality in high-acuity septic patients with high plasma IL1RA [180]. Additionally, Huang et al. have summarized several pharmacological interventions to rejuvenate HSCs, such as rapamycin, CASIN, TN13, SB203580, and ABT263 [181]. Notably, rapamycin, as an mTOR inhibitor, can enhance the regenerative capacity of HSCs. Evidence from clinical trials has demonstrated that mTOR inhibitors could improve immune function and reduce infections in older adults [182, 183]. As mentioned in “Immunosenescence in young patients with sepsis”, a single inflammatory challenge could induce the aging of HSCs, it is advisable to initiate treatments aimed at rejuvenating HSCs at the early stage of sepsis.

### Suppress the excessive inflammatory responses induced by aged immune cells

Since aged immune cells, especially aged neutrophils, play a critical role in excessive inflammation, targeting these cells might be a promising treatment. Moreover, given that early-stage sepsis is typically characterized by a sustained hyperinflammatory response, such interventions would be most effective when administered at an early time point.

#### Reduce the quantity of aged neutrophils and improve their immune functions

Preventing the formation of prematurely aged neutrophils or promoting the apoptosis of neutrophils will control the quantity of aged neutrophils. Furthermore, targeting senescent neutrophils exhibiting rTEM could ameliorate inflammatory lesions of remote organs.

Firstly, inhibition of TLRs-mediated signaling pathways could prevent sepsis-related aberrant aging of neutrophils. As mentioned above, accelerated aging of neutrophils could be induced via the activation of TLRs and Mdy88-mediated signaling pathways during sepsis. Therefore, the suppression of these pathways might attenuate aberrant aging and mitigate aggravated inflammation. For example, multiple researches have confirmed that pharmacological inhibition of TLR4, such as TAK-242 [184, 185], C34 [186], or eritoran tetrasodium (E5564) [187], leads to significant improvement in septic models. Additionally, Kim et al. have proven that fatty acid synthase (FASN)-induced Mdy88 palmitoylation is required for TLRs-mediated inflammation. Therefore, the usage of FASN inhibitor, C75, improves the survival of CLP mice via the suppression of inflammation [188]. Moreover, T-cell lymphopenia, which occurs in the early stage of sepsis, is responsible for the severe inflammation due to TLR activation. And the administration of soluble CD4 (sCD4) could attenuate innate inflammatory responses [189]. However, the results of clinical trials indicate that TLR inhibitors do not show significant impacts on the mortality of septic patients. For example, TAK-242 fails to suppress the levels of cytokines in patients with severe sepsis. Although lower mortality is observed in treated group, the difference is not significant [190]. Another TLR4 antagonist E5564 is associated with lower mortality in severe sepsis patients in phase II clinical trials [191, 192]. However, the result of multinational phase III trial demonstrates that the usage of E5564 can not reduce 28 day mortality in sepsis [193]. Since accumulating evidence suggests that single inhibition of TLRs might not be sufficient to improve the prognosis of patients [194], combination treatments have emerged as a promising approach. Combined suppression of complement C5 and CD14 (a coreceptor for several TLRs) can mitigate inflammation more effectively [195, 196]. Although a variety of TLR inhibitors have been used to verify their anti-inflammatory properties, there are few researches exploring the correlations between accelerated aging of immune cells and TLR inhibitors. Exploring the dosage of TLR inhibitors or combination therapy regimen from the perspective of premature aging might be more promising.

Secondly, promoting neutrophil apoptosis could prevent the persistent existence of aged neutrophils. It has been recognized that defective apoptosis increases aged neutrophils in the circulation due to prolonged lifespan [71]. Therefore, promoting neutrophil apoptosis may alleviate senescent neutrophil-induced persistent inflammation. Since excessive apoptosis in non-immune cells [197] or adaptive immune cells [198] will aggravate organ damage or induce immunosuppression, it would be better to in situ selectively target aged neutrophils. Recently, many researchers have successfully constructed several vehicles that help to selectively promote neutrophil apoptosis, such as natural glycyrrhiza protein nanoparticles loaded with dexamethasone [199], doxorubicin (DOX)-conjugated protein nanoparticles [200], or mesenchymal stem cell (MSC)-derived apoptotic vesicles [201]. Additionally, upregulated PD-L1 has been found in neutrophils during sepsis [202] or in senescent neutrophils [203], which can delay neutrophil apoptosis and further promote organ damage. Therefore, the suppression of PD-L1 expression in neutrophils might be a potential therapy to attenuate excessive inflammation. Moreover, inhibition of PD-L1 could further prevent a shift to immunosuppressive state during sepsis [204]. Additionally, pharmacological inhibition of excessive NET release, such as PAD4 inhibitor [205] or DNase [206], could also mitigate inflammatory responses and prevent the differentiation from CD4^+^ T cell to Treg [80]. A clinical trial has suggested that the treatment of long-acting nanoparticulate DNase-1 could decrease cfDNA levels and suppress neutrophil activities [207]. Moreover, the Second Multicentre Intrapleural Sepsis Trial (MIST2) has demonstrated that combined tissue plasminogen activator (t-PA) and DNase treatment exhibits notable therapeutic efficacy and is highly cost-effective [208].

Thirdly, suppressing rTEM of aged neutrophil subset might mitigate aggressive inflammation. Both sepsis-induced premature aging neutrophil subset in young individuals and senescent neutrophils in old sepsis patients exhibit rTEM. Multiple factors during sepsis, such as endothelial cell-derived EVs [78], CIRP [209], elevated levels of chemokines in the circulation induced by vascular leakage [210], drive aberrant neutrophil migration and ultimately cause remote organ injury. Thus, inhibiting these factors might alleviate abnormal migration. Moreover, increased CXCL1 in EC junctions is reported to drive neutrophil rTEM in old mice, and thereby CXCL1 blockade could protect aged mice from inflammation-related organ damage [91]. Additionally, a randomized controlled trial suggests that high-dose simvastatin enhances the accuracy of neutrophil migration [88]. Since various inflammatory mediators are responsible for neutrophil abnormal migration, exploring the specific target in the aged neutrophil subgroup exhibiting rTEM might be a potential therapeutic strategy.

It is worth noting that the immunological roles of aged neutrophils remain controversial, possibly due to the existence of distinct subpopulations within this group or other yet-unclear complexities. Current therapeutic strategies primarily aim to suppress their number and function during the early stage of sepsis. A recent study published in 2025 demonstrates that senescent neutrophil-derived vesicles could promote the resolution of inflammation during the later stage of the disease [211]. Further interventions targeting senescent neutrophils in late-stage sepsis need to be explored in the future.

#### Induce the transition from a senescent pro-inflammatory phenotype to an anti-inflammatory phenotype of macrophages

As mentioned above, decreased ratio of NAD^+^/NADH is closely associated with cellular senescence [212] and induces macrophages to exhibit a pro-inflammatory phenotype [213]. Thus, several studies have demonstrated that direct administration of NAD^+^ or NMN (a precursor of NAD^+^) could trigger macrophage reprogramming and further attenuate inflammation during sepsis [214–216]. Moreover, other drugs, such as cichoric acid (CA), also ameliorate sepsis-induced organ damage via regulating NAD^+^/NADH ratio [217, 218]. Additionally, blockade of NADase CD38 on macrophages is reported to mitigate inflammation [219]. And the activation of Sirtuins (the family of NAD^+^-dependent histone deacetylases), such as Sirtuin 1 (Sirt1) [220], Sirt3 [221], or Sirt6 [222], induces M2 polarization of macrophages, thereby alleviating multiple organ injury. A clinical trial has demonstrated that the treatment with a mixture of combined metabolic activators (CMAs), comprising NAD^+^ precursors and glutathione, administered during the early stage of the disease (day 1–day 14), could result in a more rapid symptom-free recovery in mild-to-moderate COVID-19 [223]. Additionally, several clinical trials have confirmed that treatment with NAD^+^ suppresses inflammatory activation and reduces cellular senescence among other inflammatory diseases [224–226]. Pretreatment of SRT2104, selective Sirt1 activator, has also been proven to attenuate LPS-induced elevation of cytokines in humans [227]. Collectively, clinical trial findings suggest that interventions aimed at inducing the anti-inflammatory phenotype of macrophages, including NAD^+^ supplement and Sirt1 activation, should be implemented during the initial phase of the disease to ensure effective suppression of the inflammatory response. Notably, TREM2 expression level is reported to decrease in elderly sepsis patients [120], and TREM2 deficiency in macrophages results in defective anti-bacterial capacity [126] and excessive inflammation [121]. Therefore, triggering TREM-2 could enhance bacterial clearance and protect against inflammatory lesions in old septic patients [120].

#### Inhibit SASP in adaptive immune cells

Since ATM-p38 axis plays a critical role in genotoxin-induced SASP in T cells [143], the usage of small-molecule inhibitor BIRB 796, which blocks p38 MAPK signaling pathway, might reverse SASP in T cells and attenuate persistent inflammation during sepsis [228]. A clinical trial has demonstrated that pretreatment of BIRB 796 could strongly inhibit LPS-induced coagulation activation [229]. Additionally, targeting the B cell-macrophage axis might mitigate macrophage-mediated inflammation in older septic patients [164].

### Prevent immunosenescence-mediated immunosuppressive state during sepsis

It would be better to improve the immunosuppressive state of sepsis from two aspects. Firstly, overactivated innate immune responses induce the shift from acute inflammation to persistent immunosuppression. Therefore, suppressing the interactions between senescence neutrophils or monocytes/macrophages and T cells might be a potential therapeutic strategy. Secondly, the treatments to directly enhance the proliferation and function of T/B cells could also improve adaptive immunity. Since immunosuppressive states primarily occur in the later stage of sepsis, relevant interventions are typically aimed at modulating immune responses and restoring immune function during this phase. However, a study has demonstrated that immune suppression occurs within 24 h in severe sepsis [230]. Increasing evidence indicates that combined treatments of anti-inflammatory drugs and immunopotentiating agents in the early stage of the disease might improve therapeutic efficacy [231].

#### Suppress the interactions between T cells and aged innate immune cells to prevent immunosuppressive state during sepsis

As mentioned above, aged neutrophils could induce T cell exhaustion and subsequent adaptive immune dysfunction. Thus, downregulating PD-L1 expression or inhibiting NET release in neutrophils could hamper the interactions between neutrophils and T cells, and thereby prevent immunosuppression in the late state of sepsis.

Pharmacological sirtuins activators can attenuate excessive inflammation, while the suppression of Sirtuins will help to reverse endotoxin tolerance in the immunosuppressive state of sepsis [232]. For example, Roger et al. demonstrate that Sirt2 deficiency restores bacterial phagocytosis of macrophages during infection [233]. And this research team has also proven that SIRT2/3^−/−^ mice are protected from endotoxemia [234]. Since current animal studies have shown that deletion of Sirtuin 2 gene restores macrophage immune functions, especially phagocytosis, it is suggested that Sirt2 inhibition strategies should be applied at an early stage of sepsis. Notably, these findings also indicate functional differences among proteins in Sirtuin family. Additionally, PD-L1 expression is increased in monocytes and B cells, and PD-L1 blockade can reverse the dysfunction of monocytes and partially suppress lymphocytes apoptosis [235]. Currently, clinical trials investigating the immunomodulatory effects of anti-PD-L1 therapy are primarily focused on cancer [236, 237], and further clinical validation is required in sepsis. Drugs that suppress cGAS-STING signaling pathway may also prevent the immunosuppressive state during sepsis, as activation of this pathway induces a senescent phenotype of macrophage, and the released SASP-related cytokines further promote T cell senescence [14]. Specifically, the administration of ALDH-2 [238], HET0016 [239], RU.521 or H-151 [240], protects sepsis-associated multiple organ damage via the inhibition of cGAS-STING signaling pathway.

#### Improve the proliferation and immune function of T/B cells to enhance adaptive immunity

In addition to the interactions between T cells and other immune cells which contribute to defective adaptive immunity, sepsis-induced thymus involution or atrophy also result in T cell senescence and immunosuppression [146]. A study has demonstrated that the administration of 4- [[4-(4-Chlorophenyl)-2-thiazolyl]amino]phenol (SK I-II), which inhibits S1P, could prevent thymus atrophy and further promote lymphocytosis [149]. Additionally, multiple studies have suggested that treatments to suppress lymphocyte apoptosis or promote their proliferation, such as decoy receptor 3 (DcR3) [241], baicalin [242], anti-LAG-3 antibody [243], could effectively alleviate immunosuppression. Moreover, the inhibition of HMGB1-PTEN signaling pathway is reported to restore the proliferation and function of T cells [244]. The usage of IgM-enriched intravenous immunoglobulins might help to improve adaptive immune function due to the insufficient production of IgM in elderly patients with sepsis [165, 245]. IgM-enriched immunoglobulin treatment, which is initiated on the day of sepsis diagnosis and continues for 3 days, failed to show beneficial effects on mortality rate in patients with severe sepsis [246]. However, the treatment with trimodulin (polyvalent IgM, IgA, IgG solution) has been proven to modify the dysregulated inflammatory response more rapidly in a phase II trial [247].

## Conclusions and future perspectives

With the development of technology, such as flow cytometry, bulk sequencing (bulk-seq), or scRNA-seq, mounting evidence suggests that inflammatory exposure during sepsis drives the expansion of immune cell subset exhibiting accelerated aging. In young septic individuals, these aged innate immune cells might be equipped with enhanced anti-bacterial capacity, however, excessive activation of aged neutrophils or macrophages could further induce inflammatory cytokine storm. Persistent activation of innate immunity might contribute to the exhaustion and the release of immature immune cells into the circulation. Meanwhile, these aged cells have been reported to interact with T/B cells, which might further impair adaptive immune responses. Moreover, severe infections can also directly induce the premature aging of T cells and further result in an immunosuppressive state. It is notable that there is no obvious boundary between acute inflammation stage and immunosuppressive state during sepsis, since pro-inflammatory and anti-inflammatory factors are both activated at the initial stage of disease.

In aged septic patients, the synergistic effects of both sepsis and aging may cause more considerable immunosenescence-related alterations. Therefore, these elderly patients could exhibit more excessive innate immune responses and more defective adaptive immune function, both of which contribute to a worse prognosis. Of note, there are differences between aberrant aged immune cell subsets in young septic individuals and the aged immune cells isolated from old patients, especially neutrophils. We speculate that senescent neutrophil subset could be further subdivided by their expressions of immune function-related genes. And aged neutrophil subgroup with decreased phagocytosis-related genes and increased rTEM-related genes accounts for a larger proportion in old sepsis patients. Therefore, the phenotype and function of aged immune cell subsets need more explorations.

Nowadays, multiple researches have suggested that different stages of sepsis require different therapeutic strategies. For example, pharmacological activation of SIRT1 has been shown to suppress the production of inflammatory cytokines during the hyper-inflammatory stage. Conversely, the inhibition of SIRT2 reverses endotoxin tolerance and enhances immune function. As mentioned above, the boundary of the two stages during sepsis is blurred, since pro-inflammatory and anti-inflammatory responses are both activated at the beginning. Therefore, several clinical trials have proven that the combined treatments of anti-inflammatory drugs and immunopotentiating agents, such as urinary trypsin inhibitor (UTI) ulinastatin + thymosin alpha1 (Tα1), could improve the prognosis of sepsis patients [231, 248]. Of note, current treatments are mainly confined to targeting immune status of patients. Since immunosenescence occurs in sepsis patients from different age classes and might be correlated with different immune statuses throughout the entire disease, the treatment targeting immunosenescence is promising. For example, thymus regeneration or IL-7 administration has been reported to improve the aging of immune system in the elderly, and these treatments could be tested in septic models or patients in the future [249, 250].
